# Wheat silage inclusion levels as a substitute for corn silage in diets of lactating Holstein × Gyr crossbred cows

**DOI:** 10.1007/s11250-026-04930-0

**Published:** 2026-03-02

**Authors:** Luciano Luís Jacob, Rafael Monteiro Araújo Teixeira, Edilane Aparecida da Silva, Angelo Herbet Moreira Arcanjo, Maurício Antônio de Oliveira Coelho, Jessica Ewilin de Sousa, Taiane Sena Santos, Clenderson Corradi de Mattos Gonçalves, Marcio de Souza Bastos, Valdir Botega Tavares, Marcela Queren Ribeiro da Cunha, Helder Felipe Cruz do Nascimento

**Affiliations:** 1 Departamento de Acadêmico de Zootecnia, Instituto Federal do Sudeste de Minas Gerais - Campus Rio Pomba, Rio Pomba, Minas Gerais Brazil; 2 Empresa de Pesquisa Agropecuária de Minas Gerais, EPAMIG Sudeste, Viçosa, Minas Gerais Brazil; 3 Empresa de Pesquisa Agropecuária de Minas Gerais, EPAMIG Oeste, Campo Experimental de Sertãozinho, Patos de Minas, Uberaba, Minas Gerais Brazil; 4 Empresa de Pesquisa Agropecuária de Minas Gerais, EPAMIG Sudeste, Campo Experimental de Leopoldina, Leopoldina, Minas Gerais Brazil; 5 Faculdade de Medicina Veterinária, Universidade Federal do Recôncavo Baiano, Cruz das Almas, Bahia Brazil; 6 Empresa de Pesquisa Agropecuária de Minas Gerais, EPAMIG, Belo Horizonte, Minas Gerais Brazil; 7 Departamento de Agronomia, Instituto Federal do Triângulo Mineiro - Campus Uberaba, Uberaba, Minas Gerais Brazil; 8 Instituto de Zootecnia, Agência Paulista de Tecnologias do Agronegócio, Ribeirão Preto, São Paulo, Brazil

**Keywords:** Apparent digestibility, Feeding behavior, Intake, Milk production

## Abstract

Tropical wheat cultivars can serve as an alternative to corn silage as a second-crop forage in the Cerrado region. This study aimed to evaluate the intake, digestibility, milk performance, and feeding behavior of crossbred Holstein × Gyr dairy cows fed diets containing increasing levels of wheat silage as a replacement for corn silage. Twelve cows with 518 kg body weight and 10.89 kg/day of milk production were used. The animals were distributed in three 4 × 4 Latin squares, balanced according to lactation stage. Treatments consisted of four levels of wheat silage replacing corn silage on a dry matter (DM) basis: 0%, 33%, 67%, and 100%. Intakes of DM, organic matter, and neutral detergent fiber were higher (*P* < 0.05) in cows fed diets containing 0% and 33% wheat silage. Cows fed 0% wheat silage had higher acid detergent fiber intake than cows fed wheat silage (*P* < 0.05). DM digestibility, nutrient digestibility, milk production, and feed efficiency did not differ (*P* > 0.05) between treatments. The diet with 100% wheat silage inclusion resulted in higher concentrations of milk urea nitrogen (*P* < 0.05). Feeding behavior was not affected (*P* > 0.05) by wheat silage inclusion levels. Wheat silage can replace or partially replace corn silage in diets for crossbred dairy cows without compromising milk production or composition.

## Introduction

Milk production in Brazil is primarily based on pasture-based systems with supplementation. The dairy cattle herd is largely composed of crossbred cows, resulting from the cross between Holstein (*Bos taurus taurus* L.) and Dairy Gyr (*Bos taurus indicus* L.) breeds. These crossbred cows show good adaptability to tropical and subtropical climates and achieve satisfactory production levels on cultivated pastures with tropical grasses of African origin (Valle et al. 2009; Jank et al. 2014; Gomide et al. 2024).

Although pasture is the basis for milk production in the main dairy producing regions of Southeastern Brazil, this region experiences a distinct rainy season during spring and summer, and a dry season from autumn through winter, which can last from 3 to 5 months, depending on the specific location (Alvares et al. 2013). This seasonal pattern forces producers to develop forage planning strategies to supplement the herd during the dry season.

Among the strategies used to meet the nutritional needs of dairy cattle during the dry period, ensiling is prominent, particularly using corn (*Zea mays* L.) as the main forage crop. Most farmers implement two cropping seasons: the main season from spring to summer, and a second crop, known as the “off-season,” from late summer to autumn. However, the off-season is more susceptible to climatic risks, such as dry spells (characterized by weeks of hot and dry weather) at the beginning of the season or the premature onset of the dry season, which are not uncommon (Costa et al. 2019; Fabris et al. 2023).

Wheat (*Triticum aestivum* L.) can serve as an alternative forage, as already practiced by livestock farmers in southern Brazil, where the climate is humid and cold during winter (Bartmeyer et al. 2011; Quatrin et al. 2017; Scherer et al. 2024). Tropical wheat lines could also serve as a viable alternative to corn silage in regions where wheat grain is already produced. Due to their early growth cycle, they reduce the risk of yield loss toward the end of the dry season, especially in the Cerrado (tropical savanna) region (Pereira et al. 2019).

The MGS-3 Brilhante wheat cultivar was developed by Agricultural Research Company of Minas Gerais (EPAMIG) for cultivation primarily in the Cerrado biome (tropical savanna) due to its tolerance to high temperatures, resistance to lodging, and classification as a bread wheat variety (Fronza 2005). Recently, the MGS-3 Brilhante wheat cultivar has been selected for silage production during the corn off-season, as it requires only 40% of the water required by corn and has a later planting window, from March 1 to April 15 (Coelho et al. 2021). Second-crop corn is grown under rainfed conditions from January to the end of February (Pereira Filho et al. 2010). In irrigated agricultural systems, wheat can be cultivated later in the Cerrado region as a third (winter) crop, typically following soybeans (*Glycine max* L. Merrill) and corn (Pereira et al. 2019; Antonini et al. 2024; Oliveira et al. 2025).

The wheat cultivar MGS-3 Brilhante has the advantage of not having awns on the ear, which could cause lesions in the oral and intestinal mucosa of animals (Karren et al. 1994). Furthermore, the MGS-3 Brilhante cultivar is indicated for use in silage because it presents high forage yield, reaching up to 50 t of green matter/ha under irrigation during the Cerrado winter (Coelho et al. 2021). As the first forage wheat developed for tropical and subtropical climates, it represents a safer silage alternative than second-crop corn. In this context, the objective of this study was to evaluate intake, digestibility, milk yield, and feeding behavior of Holstein × Gyr (*Bos taurus taurus* × *Bos taurus indicus*) crossbred dairy cows fed diets containing increasing levels of wheat silage as a replacement for corn silage).

## Materials and methods

### Experimental area

The experiment was conducted at the Leopoldina Experimental Station of the EPAMIG), located in Leopoldina, Minas Gerais, Brazil. The Leopoldina Experimental Station is situated at approximately 21°28′35″ S latitude and 42°43′18″ W longitude, with an average altitude of 192 m above sea level. The region is characterized by an *Aw* (tropical savanna) climate, with a rainy season concentrated in summer and a dry period lasting three to four months in winter (Alvares et al. 2013).

The corn crop was sown conventionally (with soil disturbance) at the Leopoldina Experimental Field during the second crop (from January to April). Sowing was carried out at a spacing of 0.80 m between rows and 0.20 m between plants, resulting in a plant stand of 62,500 plants/ha. Fertilization was applied at planting with 350 kg/ha of NPK (08-28-16), and topdressing was applied 28 days after corn emergence with 250 kg/ha of NPK (30-00-20).

Wheat planting was carried out at the EPAMIG Vale do Piranga Experimental Station, located in the municipality of Oratórios, Minas Gerais. The MGS-3 Brilhante cultivar, developed by EPAMIG and recommended for rainfed cropping systems in Minas Gerais, was used (Fronza 2005). The Vale do Piranga Experimental Station is located at approximately 20°24′10″ S latitude and 42°48′58″ W longitude, with an average altitude of 477 m above sea level. The region of Oratórios has a *Cwb* climate (subtropical highland), with a rainy season during summer and three to four dry months in winter (Alvares et al. 2013).

Wheat was cultivated under irrigation (irrigation interval: once per week). The soil was prepared using conventional tillage. Sowing was performed using a 15-row grain drill with 17 cm spacing between rows, applying 250 kg/ha of NPK (8-28-16) fertilizer. The sowing depth was 2 cm, with a seeding rate of 70 to 80 seeds per meter of row, resulting in a population density of 350 to 400 seeds per square meter and a seed requirement of 140 to 160 kg/ha. At 30 days after emergence, 300 kg/ha of a 30-00-20 fertilizer was top-dressed. Pest and disease control measures were not required.

The wheat and corn plants were ensiled using a tractor-mounted forage harvester. The chopped forage was stored and transported in a forage wagon to the surface silo, where it was spread and compacted. The wheat was ensiled after reaching the milky grain stage, which occurred 80 days after sowing. The plants had an average height of 1.30 m and a dry matter (DM) content of 26%, measured in a forced-air oven. The corn was ensiled at 93 days, with an average height of 2.23 m and approximately 32% DM. No additives were used during ensiling.

Corn was planted at the Leopoldina Experimental Station using BM 3063 Pro2 (Biomatrix^®^/Embrapa) hybrid seeds. The soil was prepared using conventional tillage, and fertilization was carried out based on soil chemical analysis, following the recommendations from the *Fertilizer and Soil Amendment Guidelines for the State of Minas Gerais – 5th Approximation* (Ribeiro et al. 1999).

### Experimental design

Twelve crossbred Holstein × Gyr cows were used: eight ½ Holstein × ½ Gyr cows and four ¾ Holstein × ¼ Gyr cows. The cows had an average body weight of 518 kg, were 90 ± 45 days in lactation, and had an average milk production of 10.89 ± 2.49 kg/day. The cows were distributed into three Latin squares, balanced according to lactation stage and blood proportion. The experiment consisted of four treatments and four experimental periods (4 × 4 design), each lasting 15 days, with the first 12 days used for adaptation to the diets and the last 3 days for data collection.

The treatments consisted of increasing inclusion levels of wheat silage replacing corn silage on a dry matter (DM) basis: 0% (100% corn silage), 33%, 67%, and 100% wheat silage. Concentrate was offered at a ratio of 1 kg per 3 L of milk produced.

The cows were housed in individual pens measuring 4 × 6 m, equipped with shade nets, feed bunks, and water troughs, with *ad libitum* access to feed and water 24 h a day. Diets were provided as total mixed rations (TMR), offered twice daily after the morning and afternoon milkings.

### Sample collection and feed composition analysis

Feed was offered to allow 5 to 10% orts in order to estimate dry matter intake (DMI). Daily, the amounts of feed offered (forage and concentrate) and orts from the previous day were weighed to calculate DMI. During each experimental period, samples of feed, orts, and feces (total fecal collection) were obtained from each animal for chemical composition analysis. The bromatological composition of corn silage, wheat silage, and concentrate is presented on a DM basis in Table 1.

**Table 1:** Bromatological composition of corn and wheat silage and concentrate used in the experimental diets (percent basis)

	Corn Silage	Wheat Silage	Concentrate
Dry Matter (%)	32.84	27.79	89.33
Organic Matter (% DM)	28.11	19.97	89.89
Crude Protein (% DM)	5.39	10.02	21.10
Neutral Detergent Fiber (% DM)	49.99	36.29	18.39
Acid Detergent Fiber (% DM)	22.70	21.68	4.70
Ether Extract (% DM)	4.92	7.18	3.80
Non-fibrous Carbohydrates (% DM)	34.97	38.69	46,60

DM: Dry Matter

The samples were pre-dried in a forced-air oven at 55 °C for 72 h and ground in a Wiley-type knife mill equipped with a 1 mm screen. Subsequently, the samples were dried at 105 °C for 2 h to determine dry matter content (DM) (Detmann et al. 2021). Ash content was determined by incinerating the samples in a muffle furnace at 600 °C for 6 h (Detmann et al. 2021).

**Fig. 1:**
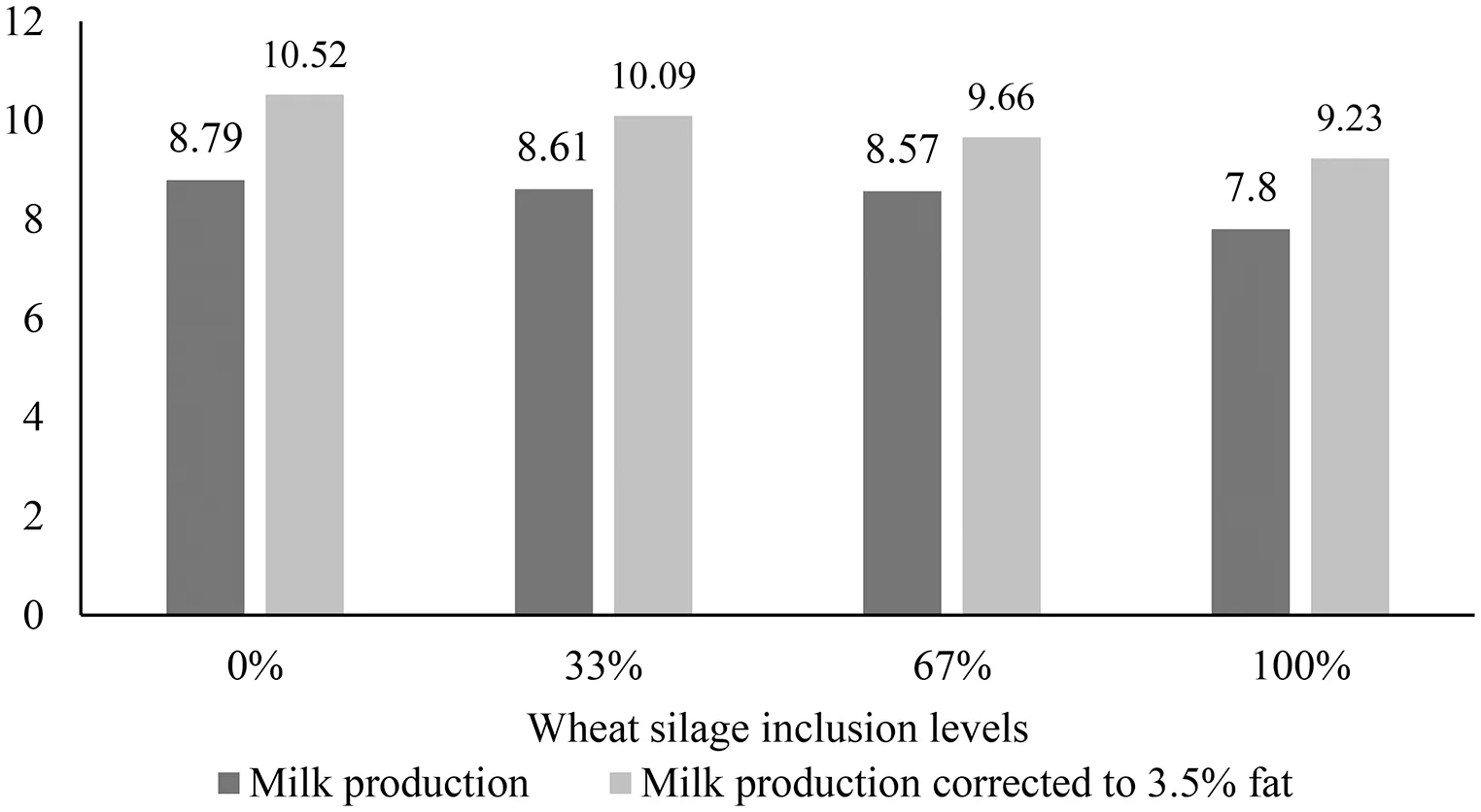
Milk production and milk production corrected for 3.5% fat of cows fed increasing levels of wheat silage replacing corn silage *MP: SEM = 0.34*,* P-value = 0.1601 | MP*_*3.5%*_: *SEM = 0.55*,* P-value = 0.2421* *SEM - standard error of the mean*

The chemical composition of the feedstuffs (Fig. 1), orts, and feces was analyzed at the Animal Nutrition Laboratory of the Federal Institute of Education, Science and Technology of the Southeast of Minas Gerais (IF Sudeste MG), in Rio Pomba, Minas Gerais, Brazil. Analyses were conducted following the standard procedures of the National Institute of Science and Technology in Animal Science (Detmann et al. 2021) for determining the contents of ash, organic matter (OM), neutral detergent fiber (NDF), acid detergent fiber (ADF), crude protein (CP), and ether extract (EE). The non-fiber carbohydrate (NFC) contents were estimated using the equation proposed by Detmann et al. (2021):

$${\rm{NFC = 100 - ash - NDF - CP - EE}}$$

### Dry matter and nutrient intake

Dry matter intake was calculated as the difference between the dry matter content of the diet offered and the dry matter content of the orts. Nutrient intake (OM, CP, NDF, ADF, EE, and NFC) was estimated by multiplying the nutrient content of the diet by the amount of dry matter consumed.

### Apparent digestibility of dry matter and nutrients

To obtain the apparent digestibility coefficients of dry matter and nutrients (OM, CP, NDF, ADF, EE, and NFC), the total fecal collection method was used according to Berchielli et al. (2005). The feces of each animal were collected over a 24-hour period on three consecutive evaluation days, corresponding to the last three days of each experimental period, using buckets adapted for this purpose; the material was individually weighed, stored in plastic bags, labeled, and frozen at -10 °C, whereas feces collected from the pen floor were only weighed and then discarded.

During each 24-hour collection period, the fecal samples were homogenized to form composite samples of approximately 500 g, totaling three samples per experimental period, which were then pre-dried in a forced-air oven at 55 °C for 72 h and ground in a Wiley-type knife mill with a 1.0 mm screen for bromatological analyses at the Animal Nutrition Laboratory of IF Sudeste MG (Detmann et al. 2021). The apparent digestibility of DM (DigDM) and nutrients (DigNut) was determined using the equations proposed by Van Soest (1994) and expressed as a percentage:

$$\:DigDM\:=\:\frac{DM\:intake\:(DM\:fecal\:-\:CE)\:}{DM\:intake}$$

$${\rm{CE = (0}}{\rm{.098 \div MS intake)}}$$

$$\>DigNut\> = \>\left[ {{\matrix{ (DM\>intake\> \times \>\>\% \>Nutrient)\> \hfill \cr - \>(DM\>excreted\> \times \>\>\% \>Nutrient)\> \hfill \cr} \over {(DM\>intake\> \times \>\>\% \>Nutrient)}}} \right]$$.

### Milk production, milk quality, and feed efficiency

The cows were mechanically milked twice a day, and milk production (MP) was recorded. A device attached to the milking machine measured the amount of milk produced per cow, and milk samples were collected on the last two days of each experimental period (14th and 15th days).

Milk samples were stored in plastic containers containing preservative (Bronopol^®^) and maintained at 2 to 6 °C. Subsequently, they were sent to the Lactation Physiology Laboratory (Milk Clinic), Department of Animal Science, Luiz de Queiroz College of Agriculture (ESALQ/USP), in Piracicaba, São Paulo, for analysis of milk components (protein, fat, lactose, total solids, and solids-not-fat) and milk urea nitrogen. These variables were determined by mid-infrared spectrometry, according to PO ANA 001:06 (IDF ) (Sklan et al 1992).

The MP values for each cow were corrected to 3.5% fat (MP_3.5%_) according to the equation proposed by Sklan et al. (2012):

$$\eqalign{ {\rm{M}}{{\rm{P}}_{{\rm{3}}{\rm{.5\% }}}}{\rm{ = }} & {\rm{ }}\left( {{\rm{0}}{\rm{.432 + 0}}{\rm{.1625 \times \% milk}}\,{\rm{ fat}}} \right){\rm{ + }} \cr {\rm{ }} & \left[ {{\rm{15 \times }}\left( {{\rm{fat }}\,{\rm{production \times milk}}\,{\rm{ production / 100}}} \right)} \right] \cr}$$

Feed efficiency (FE) was determined as per Silva et al. (2022), calculated as the ratio of MP/DMI and MP_3.5%/_DMI.

### Ingestive behavior

On day 12 of each experimental period, the cows’ ingestive behavior was visually assessed by four trained individuals, who rotated every four hours. Observations were made over a 24-hour period, starting after the morning milking, when the animals arrived at the trough, and ending the next day when the cows left for milking. The observations were made at 10 min intervals, during which the cows’ ingestive behavior was recorded, including feeding, ruminating, drinking, and resting (Johnson and Combs 1991).

### Statistics

The data on DMI, nutrient intake, dry matter digestibility, milk performance, milk quality, and ingestive behavior were subjected to analysis of variance in a Latin square design with regression, using the SISVAR© software (Ferreira et al. 2011). The means between treatments were compared using Tukey’s test at a 5% significance level.

## Results

### Dry matter and nutrient intake

The DMI and OM intake were higher (*P* < 0.05) in cows fed 0 and 33% wheat silage inclusion (Table 2). Cows fed 67% wheat silage inclusion had intermediate DMI and OM intakes.

**Table 2:** Dry matter and nutrient intake of dairy cows receiving wheat silage as a replacement for corn silage

Intake	Wheat silage inclusion levels					
	0%	33%	67%	100%	SEM	P-value
	*kg/day*					
Dry matter	15.95^a^	16.10^a^	13.82^ab^	13.00^b^	0.56	0.0016
Organic matter	14.79^a^	14.38^a^	12.55^ab^	11.72^b^	0.51	0.0012
Crude protein	1.42	1.61	1.50	1.62	0.06	0.1245
Neutral detergent fiber	8.05^a^	7.60^a^	5.96^b^	5.35^b^	0.40	0.0004
Acid detergent fiber	3.58^a^	3.70^ab^	3.35^ab^	2.98^b^	0.16	0.0199
Ether extract	0.73	0.74	0.71	0.66	0.06	0.7469
Non-fibrous carbohydrates	9.03	9.01	8.33	7.83	0.36	0.0768

SEM - standard error of the mean

Neutral detergent fiber intake was higher (*P* < 0.05) in cows fed lower wheat silage inclusion levels (Table 2). Cows fed 0% wheat silage inclusion had the highest ADF intake (*P* < 0.05), while 33% and 67% inclusions showed intermediate ADF intake. No significant difference (*P* > 0.05) was found for CP and EE intakes between cows fed increasing wheat silage levels (Table 2). However, there was a trend (0.05 ≤ *P* < 0.10) for lower wheat silage inclusion levels to increase NFC intake.

### Apparent digestibility of dry matter and nutrients

The inclusion of wheat silage did not influence (*P* > 0.05) the apparent digestibility of DM in the evaluated diets (Table 3). Similarly, no difference was observed in the apparent digestibility of OM, CP, NDF, ADF, EE, and NFC (Table 3).

**Table 3:** Apparent digestibility of dry matter and nutrients (%) of diets with increasing levels of wheat silage replacing corn silage

Apparent digestibility	Wheat silage inclusion levels					
	0%	33%	67%	100%	SEM	*P*-value
Dry matter (%)	59.09	60.20	61.32	62.43	4.48	0.8141
Organic matter (%)	67.45	68.10	68.74	69.39	2.53	0.2035
Crude protein (%)	66.46	67.41	68.36	69.32	3.51	0.6865
Neutral detergent fiber (%)	71.25	73.35	75.44	77.54	2.76	0.2615
Acid detergent fiber (%)	59.72	58.50	59.28	62.07	3.70	0,6814
Ether extract (%)	70.09	68.57	67.04	65.52	4.40	0.6753
Non-fibrous carbohydrates (%)	77.86	77.47	77.07	76.68	2.05	0.3201

SEM - standard error of the mean

### Milk production and feed efficiency

**Fig. 2:**
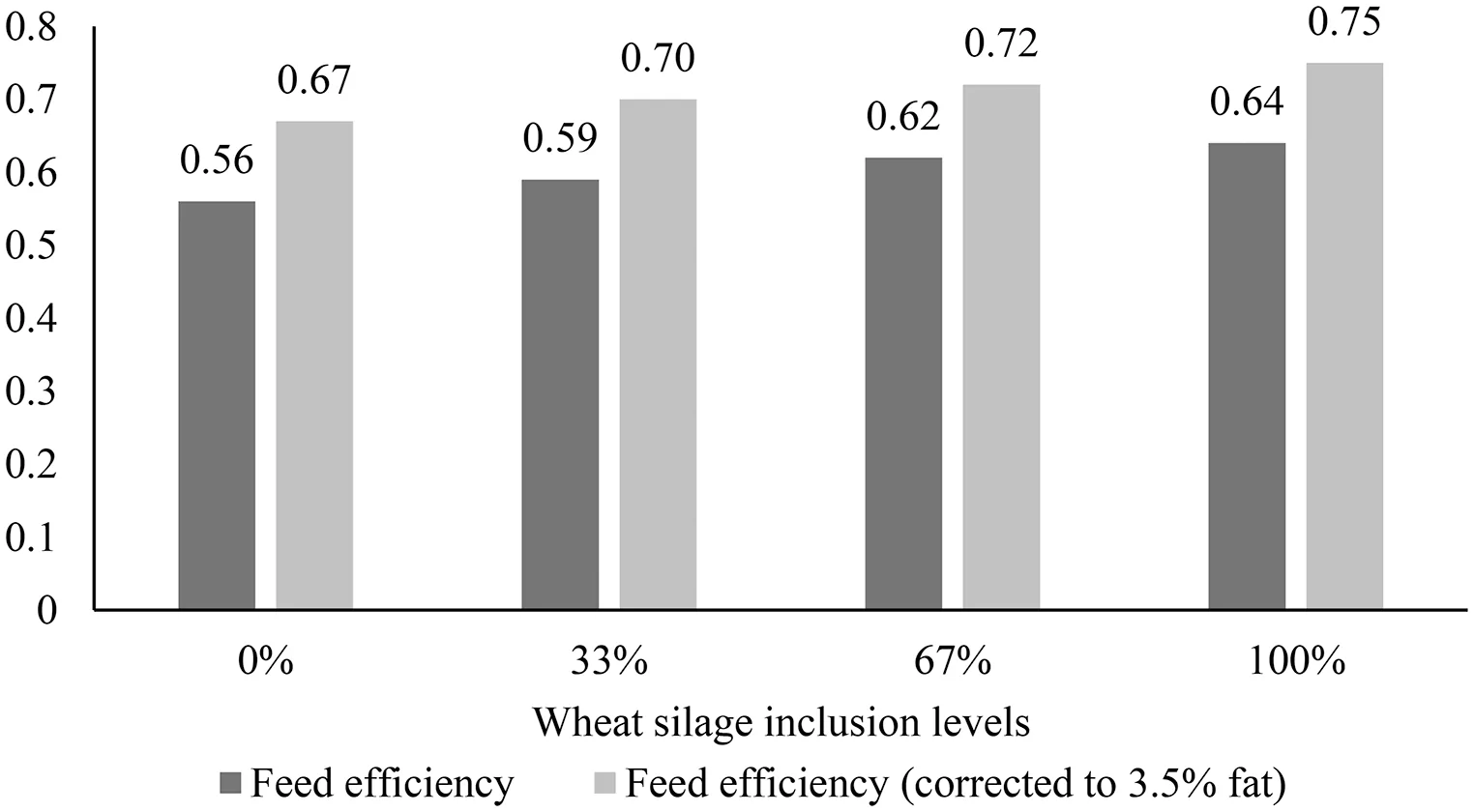
Feed efficiency and feed efficiency corrected for 3.5% milk fat of cows fed increasing levels of wheat silage replacing corn silage *FE*: *SEM = 0.04*,* P-value: 0.1416 | FE*_*3.5%*_: *SEM = 0.05*,* P-value = 0.2250* *SEM - standard error of the mean*

The wheat silage inclusion levels did not significantly affect (*P* > 0.05) MP and MP_3.5%_ among the cows, presenting averages of 8.44 and 9.88 L/cow/day, respectively (Fig. 1). Likewise, FE and FE_3.5%_ did not show any difference (*P* > 0.05) between the wheat silage inclusion levels, presenting averages of 0.60 and 0.71, respectively (Fig. 2).

### Milk composition

The inclusion of wheat silage did not influence (*P* > 0.05) the protein, lactose, total defatted solids, and total solids content in milk, presenting averages of 3.45%, 4.26%, 8.68%, and 13.31%, respectively (Table 4). There was a trend toward higher milk fat content (0.05 ≤ *P* < 0.10) in cows fed 100% wheat silage, with approximately 12% more fat than the milk from cows fed 0% wheat silage (Table 4). Milk urea nitrogen was higher (*P* < 0.05) with 100% wheat silage inclusion compared to the treatment without wheat silage inclusion, with 17.51 mg/dL versus 8.93 mg/dL, respectively.

**Table 4:** Milk composition of crossbred Holstein × Gyr cows fed increasing levels of wheat silage replacing corn silage

	Wheat silage inclusion levels					
	0%	33%	67%	100%	SEM	*P*-value
Fat (%)	4.74	4.45	4.03	5.29	0.32	0.0715
Protein (%)	3.42	3.44	3.39	3.54	0.13	0.8832
Lactose (%)	4.35	4.24	4.36	4.07	0.11	0.2528
Defatted dry extract (%)	8.78	8.65	8.70	8.59	0.09	0.6733
Total solids (%)	13.52	13.10	12.73	13.88	0.33	0.1139
Urea nitrogen (mg/dL)	8.93^b^	10.76^b^	14.59^ab^	17.51^a^	0.66	0.0001

SEM - standard error of the mean

### Ingestive behavior

No significant difference (*P* > 0.05) was observed between wheat silage inclusion levels regarding ingestive behavior variables (Table 5). The overall means for feeding, rumination, resting, drinking water, and milking were 355, 532, 309, 26, and 209 min, respectively.

**Table 5:** Ingestive behavior of dairy cows fed increasing levels of wheat silage replacing corn silage

Wheat silage inclusion levels						
	0%	33%	67%	100%		
	*minutes/day*				SEM	P-value
Feeding	343.33	351.67	352.50	371.67	11.03	0.3449
Rumination	505.00	526.67	549.17	545.83	19.91	0.3968
Idleness	332.50	329.17	284.17	290.00	17.66	0.1403
Drinking water	23.33	25.83	30.00	25.00	3.39	0.5651
Milking	213.33	206.66	208.33	207.50	3.80	0.6069

SEM - standard error of the mean

## Discussion

The reduction in DM, OM, NDF, and ADF intake by cows fed 100% wheat silage may have been due to the different compositions of corn and wheat silages (Table 1). Wheat silage contains a lower dry matter content (27.79%), is ensiled at an earlier maturity stage than corn (32.84%), and therefore has a lower NDF and ADF content. Contrary to what was observed, Álvarez-García et al. (2023) found no difference in DM intake in Holstein cows fed 0, 50, and 100% wheat silage as a replacement for corn silage. Cui et al. (2023) observed a 4%, 12%, and 27% decrease in DMI for sheep fed 36%, 64%, and 100% wheat silage replacing oat hay, compared to 0% inclusion.

The diets presented an average apparent DM digestibility of 60.76%. In agreement with the present study, Cui et al. (2023) observed no differences in apparent dry matter digestibility with increasing wheat silage inclusion (average of 78.33%). Shaani et al. (2017) found higher dry matter digestibility in cows fed wheat silage diets compared to cows fed chopped wheat hay and wheat hay with long particles (65.2%, 61.8%, and 62.4%, respectively).

Because corn is a C_4_ grass, the apparent digestibility of NDF and ADF was expected to be lower due to its thicker cell wall than that of C_3_ grasses, such as wheat, which hinders degradability by rumen microorganisms (Carvalho and Pires 2008; Taiz et al. 2022). This did not influence the apparent digestibility of NDF and ADF with increased inclusion of wheat silage as a substitute for corn silage (averages of 74.40% and 59.89%, respectively). The same was observed in the apparent digestibility of CP, EE, and NFC, even though wheat was ensiled with a lower DM content than corn (Table 1), with no difference with increased inclusion of wheat silage.

Milk production and MP_3.5%_ were 8.44 and 9.87 L, respectively, for cows fed increasing wheat silage inclusion levels. Similarly, Álvarez-García et al. (2023) found no difference in milk production of Holstein cows fed diets with 0, 50, and 100% wheat silage replacing corn silage.

Although DM intake decreased with the increased inclusion of wheat silage, this did not negatively affect FE and FE_3.5%_. Silva et al. (2022) observed that the increased DM intake of cows fed fibrous co-product from sweet corn cob caused lower FE, compared to cows fed corn silage.

Wheat silage had a higher protein concentration than corn silage (Table 1). This resulted in greater milk urea nitrogen excretion in cows fed wheat silage. It is noteworthy that, in almost all treatments, urea nitrogen levels remained within the normal range for lactating cows (Roland et al. 2014).

In this study, cows received concentrate based on milk production, 1 kg of concentrate for every 3 kg of milk produced, regardless of treatment. This differs from Álvarez-García et al. (2023), who provided 4.6 kg of concentrate/cow/day to Holstein cows fed diets containing 0%, 50%, and 100% wheat silage replacing corn silage. These authors found no differences in fat, protein, lactose, and milk urea nitrogen content among the diets evaluated. This difference in the proportion of concentrate based on milk production in this study may have influenced the higher fat and milk urea nitrogen content in cows fed 100% wheat silage.

Despite the different morphological and structural characteristics of the forages, the particle density of corn silage and wheat silage did not influence ingestive behavior, especially the time spent feeding and ruminating. NDF in forage, which is less digestible, increases rumination time per kg of DMI and NDF ingested by ruminants (Beauchemin et al. 2018).

Although Shaani et al. (2017) found equal concentrations of physically effective fiber (peNDF) in long hay than in wheat silage (45.8% and 48.6% of DM, respectively), Holstein cows fed the wheat silage diet had about an hour more rumination time than cows fed the long wheat hay diet. Arcanjo et al. (2024) observed that Nelore bulls fed corn silage spent more time ruminating and less time feeding and drinking compared to bulls fed cottonseed cake. The authors noted that, despite cottonseed cake having higher peNDF, corn silage had more particles retained on the sieve ≥ 19 mm, which stimulated longer rumination times.

The MGS3 Brilhante wheat cultivar is tolerant to drought and hot weather and is suitable for silage production in areas that were idle during the winter (EPAMIG 2024). Wheat silage produced in the corn silage off-season provided efficient diets for crossbred Holstein × Gyr cows and can be offered as a total or partial replacement for corn silage.

## Conclusion

Although increased wheat silage intake reduced dry matter intake, it did not affect nutrient digestibility, ingestive behavior, milk production, and feed efficiency. Therefore, wheat silage can replace or complement corn silage in the diets of Holstein × Gyr crossbred cows without negative effects during lactation.

## Data Availability

The data supporting the findings of this study are available from the corresponding author upon reasonable request.
